# Monitoring SARS-CoV-2 Viral Load and CD4+ T-cell Count After ART in a Patient Diagnosed With AIDS Following SARS-CoV-2 Infection: A Case Report

**DOI:** 10.7759/cureus.51189

**Published:** 2023-12-27

**Authors:** Yasuhiro Umekage, Mayumi Hatayama, Akari Yagita, Kiichi Nitanai, Hiraku Yanada, Ryota Shigaki, Yoshinori Minami, Takaaki Sasaki

**Affiliations:** 1 Department of Infection Control, Asahikawa Medical University Hospital, Asahikawa, JPN; 2 Division of Metabolism and Biosystemic Science, Gastroenterology, and Hematology/Oncology, Department of Internal Medicine, Asahikawa Medical University, Asahikawa, JPN; 3 Division of Respiratory Medicine and Neurology, Department of Internal Medicine, Asahikawa Medical University, Asahikawa, JPN

**Keywords:** pneumocystis pneumonia, hiv, acquired immune deficiency syndrome (aids), aids, sars-cov-2

## Abstract

We describe the case of a 36-year-old man diagnosed with human immunodeficiency virus (HIV) following prolonged severe acute respiratory syndrome coronavirus 2 (SARS-CoV-2) pneumonia. The patient had a complication of pneumocystis pneumonia. Upon initiating highly active antiretroviral therapy, we monitored HIV RNA levels, CD4+ T-cell count, SARS-CoV-2 viral load, and IgG antibodies against SARS-CoV-2. At 167 days post SARS-CoV-2 diagnosis, the patient’s CD4+ T-cell count increased to 180 cells/μL. IgG antibodies against SARS-CoV-2 were undetectable despite a decreased SARS-CoV-2 viral load. HIV screening is necessary in cases of prolonged SARS-CoV-2 pneumonia or persistent SARS-CoV-2 shedding. When diagnosed with HIV infection, it is advisable to consider the possibility of pneumocystis pneumonia.

## Introduction

Human immunodeficiency virus (HIV) infection leads to a gradual reduction in CD4+ T cells, resulting in diminished cellular immunity and progression to acquired immune deficiency syndrome (AIDS). Patients with HIV and a CD4+ T-cell count <350 μ/L are at an elevated risk for severe acute respiratory syndrome coronavirus 2 (SARS-CoV-2) infection [1]. Antiretroviral therapy (ART) plays a pivotal role in preventing the depletion of CD4+ T cells in HIV-infected individuals. In this case report, we highlight the observed decrease in the SARS-CoV-2 viral load and concurrent increase in the CD4+ T-cell count in a patient with AIDS after the initiation of ART.

## Case presentation

**Figure 1:**
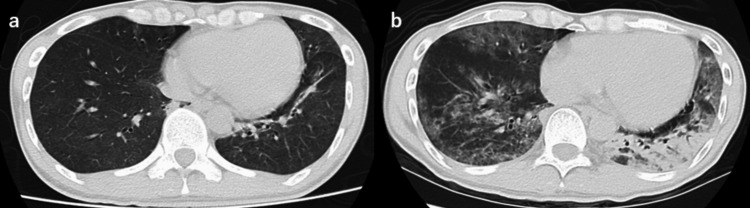
CT scan of the chest. (a) At eight days after the diagnosis of SARS-CoV-2 infection, diffuse ground-glass opacities are observed. (b) At 24 days after the diagnosis of SARS-CoV-2 infection, diffuse ground-glass opacities and infiltrative shadows in the left lower lobe are observed. CT: computed tomography; SARS-CoV-2: severe acute respiratory syndrome coronavirus 2.

A 36-year-old male presented to a local clinic with a fever. He had no history of receiving the SARS-CoV-2 vaccine. He tested positive for the SARS-CoV-2 antigen and received antipyretic treatment. However, eight days post-diagnosis, the patient was hospitalized due to respiratory failure. Chest computed tomography (CT) revealed pneumonia (Figure 1). He underwent treatment with molnupiravir (1.6 g/day) for five days and dexamethasone (6.6 mg/day) and ceftriaxone (2 g/day) for 10 days. His respiratory condition improved, and he was discharged 23 days after the diagnosis of the SARS CoV-2 infection.

**Figure 2:**
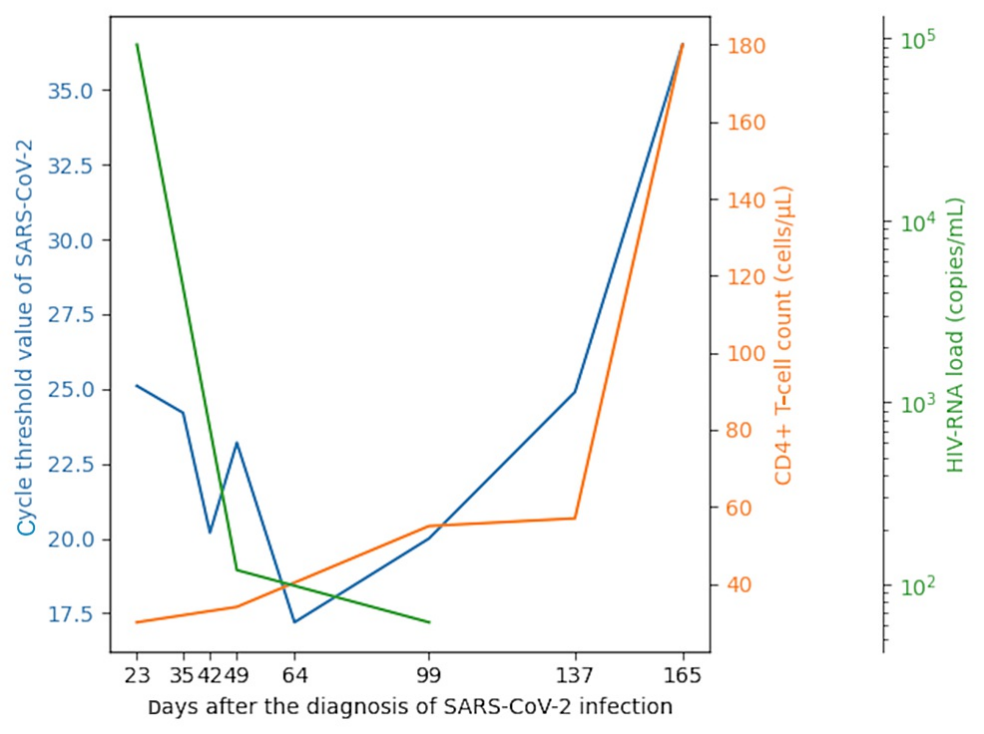
Changes in the cycle threshold value of SARS-CoV-2 viral load, HIV-RNA load, and CD4+ T-cell count. The graph illustrates the changes in cycle threshold value of SARS-CoV-2, HIV-RNA load, and CD4+ T-cell count. On day 137 after the diagnosis of SARS-CoV2 infection, the HIV-RNA load became undetectable below the sensitivity threshold. On day 165 after the diagnosis of SARS-CoV-2 infection, the patient’s CD4+ T-cell count increased to 180 cells/μL, with a decrease in the SARS-CoV-2 viral load, observed with a cycle threshold value of 36.5. SARS-CoV-2: severe acute respiratory syndrome coronavirus 2; HIV: human immunodeficiency virus.

The following day, the patient experienced intensified dyspneic symptoms, prompting his transfer to our hospital. A subsequent chest CT scan revealed extensive pneumonia, necessitating readmission due to worsening respiratory failure. Upon admission, vital signs were as follows: a respiratory rate of 21 breaths/min, temperature of 38.1°C, and oxygen saturation at 98% (with a 5 L/min oxygen mask). - The treatment regimen for SARS-CoV-2 pneumonia included remdesivir (200 mg on the first day and 100 mg from day 2 to day 10), dexamethasone (6.6 mg/day) for 10 days, followed by a gradual taper, and tocilizumab (8 mg/kg) for two days. Lab tests also revealed positive results for HIV antigen/antibody and β-D-glucan (Table 1). The patient had no known risk factors for HIV infection. These results led to a diagnosis of pneumocystis pneumonia, and treatment with sulfamethoxazole-trimethoprim was initiated. The elevated HIV-RNA level (9.4 × 10^4^ copies/mL) combined with a decreased CD4+ T-cell count (30 cells/µL) confirmed the diagnosis of AIDS (Figure 2).

**Table 1:** Laboratory findings of admission to our hospital. Results of laboratory tests. Elevated levels of HIV antigen/antibody and β-D glucan are observed. Neut: neutrophils; Lymp: lymphocytes; Eos: eosinophils; AST: aspartate aminotransferase; ALT: alanine aminotransferase; Cr: creatinine; LDH: lactate dehydrogenase; CRP: C-reactive protein; HIV: human immunodeficiency virus.

Hematology	Value	Reference range
WBC (/μL)	15,380	3500-9000
Neut (%)	79.4	40-75
Lymp (%)	14	18-50
Eos (%)	1.4	0-8
Biochemistry		
AST (U/L)	23	13-40
ALT (U/L)	30	5-45
Cr (mg/dL)	0.61	0.61-1.04
LDH (U/L)	224	124-222
Serology		
CRP (mg/dL)	19.9	0-0.3
Beta-D-glucan (pg/mL)	308	0-10
KL-6 (U/mL)	2910	0-500
HIV antigen/antibody (S/CO)	1235	0-1
IgG (mg/dL)	1041	870-1700
IgM (mg/dL)	163	33-190

His respiratory condition continued to improve, and ART, consisting of raltegravir potassium (800 mg/day) and one tablet of emtricitabine and tenofovir alafenamide, was initiated 32 days after the diagnosis of SARS-CoV-2 infection. The patient was discharged on the 46th day post-diagnosis of SARS-CoV-2 infection. Subsequent SARS-CoV-2 PCR testing after discharge showed a cycle threshold value of 17.2 on the 64th day post-diagnosis. However, by the 137th day post-diagnosis of SARS-CoV-2 infection, the HIV RNA level was undetectable, falling below the sensitivity threshold. On the 165th day post-diagnosis of SARS-CoV-2 infection, CD4+ T-cell count reached 180 cells/µL, and additional PCR testing for SARS-CoV-2 revealed a reduced viral load. Concurrent assessments for IgG antibodies against SARS-CoV-2 were conducted, but the levels remained undetectable.

## Discussion

In the present case, a patient with SARS-CoV-2 infection complicated by respiratory failure was diagnosed with an HIV infection and pneumocystis pneumonia. Differentiating between SARS-CoV-2 pneumonia and pneumocystis pneumonia proved challenging based on the evident symptoms. Similar diagnostic challenges concerning these two types of pneumonia have been previously reported [2]. Jeican II et al. reported a case of co-infection with pneumocystis pneumonia in an autopsy of severe SARS-CoV-2 pneumonia [3]. Severe SARS-CoV-2 infections can also be complicated by pneumocystis pneumonia. The lung opacities observed on the CT scan of our patient did not demonstrate the rapid progression typically associated with SARS-CoV-2 pneumonia. We speculate that the observed deterioration was more attributable to pneumocystis pneumonia rather than merely contributing to the respiratory failure. The influence of COVID-19 infection on accelerating HIV infection is not clear, but there have been reports of co-infection with COVID-19 and HIV in the past. Coleman et al. reported a case of co-infection with SARS-CoV-2 infection and pneumocystis pneumonia in an HIV patient. This case report describes a well-controlled HIV patient who developed co-infection with SARS-CoV-2 infection and pneumocystis pneumonia [4]. A patient with SARS-CoV-2 infection complicated by respiratory failure was diagnosed with an HIV infection, and it should be considered that the patient is developing pneumocystis pneumonia.

In this scenario, SARS-CoV-2 shedding persisted for 165 days. There are reports of immunocompromised patients shedding SARS-CoV-2 for extended durations, with instances reaching up to 105 days, 70 of those days involve infectious shedding [5]. Prolonged shedding of SARS-CoV-2 appears to be common in HIV-infected patients with CD4+ T-cell counts below 200 cells/μL. In contrast, HIV patients with a CD4+ T-cell count of 200 cells/μL or greater exhibit a shedding duration similar to non-HIV patients [6]. In this case, IgG antibodies directed against SARS-CoV-2 were not detected. HIV viral can cause cellular immune deficiency, and as it progresses, it may also lead to dysfunction of B cell [7]. In patients with HIV with low CD4+ T-cell counts, the production of IgG antibodies diminishes even after receiving SARS-CoV-2 vaccines [8]. Pallikkuth et al. showed that in HIV-infected individuals undergoing ART who did not respond to influenza vaccination, the functionality of T follicular helper cells remained impaired [9]. Similarly in this case, the antibody response failure may persist even after initiating ART, and it is suggested the reduction in viral shedding is associated with factors other than the antibody response.

## Conclusions

In this study, we describe the case of a patient with AIDS who developed pneumocystis pneumonia after being diagnosed with SARS-CoV-2 infection. We monitored the HIV-RNA load, CD4+ T-cell count, SARS-CoV-2 viral load, and presence of IgG antibodies against SARS-CoV-2 in our patient following the initiation of ART. After observing a reduced HIV-RNA load, there was a notable increase in CD4+ T cells and a decrease in SARS-CoV-2 viral load. When pneumonia in patients with SARS-CoV-2 fails to improve or when continuous shedding of SARS-CoV-2 persists without reduction, screening for HIV infection should be considered. When diagnosed with HIV infection, it is advisable to consider the possibility of pneumocystis pneumonia.
